# Involvement of synaptophysin and microtubule-associated protein 2 in the neuroprotective effect of berberine in an amyloid β-induced rat model of Alzheimer's disease

**DOI:** 10.22038/ajp.2025.26026

**Published:** 2025

**Authors:** Mohammad-Hadi Akbarizadeh-Mashkani, Siamak Afshinmajd, Saeid Iranzadeh, Mehrdad Roghani

**Affiliations:** 1 *School of Medicine, Iran University of Medical Sciences, Tehran, Iran*; 2 *Department of Neurology, Faculty of Medicine, Shahed University, Tehran, Iran*; 3 *Neurophysiology Research Center, Shahed University, Tehran, Iran*

**Keywords:** Alzheimer’s disease, Amyloid β, Berberine, Neuroprotection

## Abstract

**Objective:**

Alzheimer’s disease (AD) is a major public health concern. Berberine has shown promise in animal models by improving memory retention through multiple mechanisms. This study aimed to evaluate berberine therapeutic potential in ameliorating cognitive deficits in a rat AD model induced by intrahippocampal amyloid β_1-42_.

**Materials and Methods:**

The AD model was induced through bilateral injection of amyloid β_1-42_ into the CA1 region of the hippocampus. Berberine was administered orally, starting one hour post-surgery for one week. Rats were divided into sham, amyloid β, amyloid β + berberine 10 mg/kg, and amyloid β + berberine 50 mg/kg groups. The assessments encompassed cognitive testing and analysis of hippocampal markers, including oxidative stress, inflammation, apoptosis, and synaptic plasticity. Additionally, we evaluated acetylcholinesterase (AChE) activity and quantified neuronal loss in the hippocampal CA1 region.

**Results:**

Berberine improved the cognitive performance of amyloid-microinjected rats in the Y-maze, novel object recognition, and passive avoidance tests in a dose-dependent manner. Berberine attenuated hippocampal levels of malondialdehyde (MDA), nitrite, and tumor necrosis factor α (TNFα). Furthermore, berberine improved the activity of superoxide dismutase (SOD) and reduced caspase-3 and AChE activity. Berberine also enhanced synaptophysin and microtubule-associated protein 2 (MAP2) levels and inhibited neuronal loss in the CA1 region.

**Conclusion:**

Berberine demonstrated protective effects against amyloid β-induced cognitive deficits in a rat AD model, and these effects were associated with reduced oxidative and nitrosative stress, inflammation, apoptosis, and AChE activity, alongside enhanced synaptic protection.

## Introduction

Alzheimer’s disease (AD) is the most common cause of dementia in older individuals, accounting for up to 80% of all dementia cases (Gustavsson et al. 2023). AD is identified by general cortex atrophy, intracellular hyperphosphorylated tau proteins, and extracellular deposition of amyloid β plaques, all contributing to neuron degeneration (Zhang et al. 2023). Other histopathological characteristics include neuronal loss of the hippocampus, neuroinflammation, dysfunction of the cholinergic system, and oxidative stress. Furthermore, aberrant dendritic and synaptic functions are correlated with cognitive impairments in AD (Rao et al. 2022). 

*Berberis vulgaris L.* is a well-established medicinal plant belonging to the Berberidaceae family and it is cultivated extensively in various regions of Asia and Europe. Phytochemical analyses of various *Berberis* species have led to the discovery and extraction of alkaloids, phenolic compounds, tannins, triterpenes, and sterols. Berberine, the primary compound present in *Berberis*, is an isoquinoline alkaloid extracted from a variety of plants, such as *Berberis petiolaris* and *B. vulgaris*. Berberine is recognized as a potent treatment for numerous chronic diseases (Gasmi et al. 2024). Berberine therapeutic action has been extensively studied in a variety of neurological disorders such as cerebral ischemia injury, Parkinson's disease, AD, and epilepsy. Studies have demonstrated that berberine can be effective in the treatment of AD through different therapeutic mechanisms (Tian et al. 2023).

Berberine increases antioxidant capacity and inhibits acetylcholinesterase (AChE) activity, amyloid β protein deposition, and tau protein hyperphosphorylation (Fang et al. 2020). Berberine reduces amyloid β pathogenesis, mitigates synapse damage, and diminishes beta-secretase 1 (BACE1) activity (Cai et al. 2018). Berberine also attenuates neuroinflammation, playing a protective role in AD treatment. Furthermore, berberine has been recognized as a protective agent against AD risk factors such as diabetes mellitus, atherosclerosis, and hypertension (Akbar et al. 2021).

This research aimed to investigate the potential of berberine in mitigating cognitive impairments induced by amyloid β in rats and to elucidate some of its mechanisms of neuroprotective action.

## Materials and Methods

### Animals

Male albino Wistar rats (200-230 gr), obtained from the Neuroscience Research Center at SBMU, were housed in the vivarium under controlled conditions at a temperature ranging from 22 to 24 ^o^C, following a 12:12 hr light-dark cycle. The rats were given unrestricted access to tap drinking water and a standard laboratory diet. All experiments were carried out in accordance with the guidelines set by the NIH for the care and utilization of laboratory animals. The research protocol obtained approval from the Ethics Committee of Shahed University, with certificate code IR.Shahed.REC.1396.102.

### Experimental groups

Forty-four rats were randomly allocated to four groups: sham, amyloid β, amyloid β + berberine 10 mg/kg, and amyloid β + berberine 50 mg/kg. In the sham group, rats underwent stereotactic surgery with intracerebral injection of normal saline (N/S 0.9%). In the remaining three groups, the AD model was induced by directly injecting the rat-specific amyloid β_1-42_ (SigmaAldrich, USA; Cat # SCP0038) into the CA1 region of Ammon's horn in the hippocampus.

Amyloid β_1–42 _was formulated as a predefined solution in sterilized 0.1 mol/L phosphate-buffered saline (PBS). The amyloid β solution (Sigma-Aldrich, USA) was aggregated by incubation at 37°C for 96 hr. The solution was injected at a concentration of 5 µg/µl (2 µl total) based on coordinates derived from the Paxinos and Watson atlas: Anteroposterior -3.5, Lateral ±2, and Depth -2.8 mm.

Anesthesia induction was performed with a combination of xylazine (10 mg/kg intraperitoneal [*i.p.*]) and ketamine (100 mg/kg *i.p.*). After shaving the surgical area and placing the head in a stereotaxic apparatus, a midline incision (~2 cm) was made to expose the skull. Following identification of the Bregma point, two symmetric holes were drilled, and amyloid β or saline was injected into the hippocampal CA1 field.

For the treatment groups, berberine hydrochloride (obtained from Santa Cruz Biotechnology, USA; Cat # sc-204645) was administered orally one hour post-surgery and continued daily for one week at doses of either 10 or 50 mg/kg per day.

**Figure 1:**
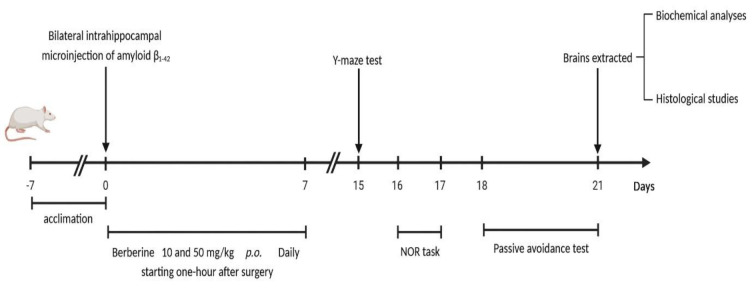
*Study d* *esign and experimental timeline*

The behavioral assessments included the Y-maze (conducted on day 15), novel object recognition (conducted on days 16–17), and passive avoidance tasks (conducted on days 18–21). At the end of the three-week postoperative period, the subjects were anesthetized using ketamine at a high dose of 150 mg/kg for subsequent biochemical and histological analyses (Baluchnejadmojarad et al. 2019). The study design and timeline are illustrated in Figure 1.

### Behavioral tasks

#### Y-maze Task

This assessment is a reliable method for assessing short-term spatial memory performance in rodents by evaluating spontaneous alternation behaviors. The apparatus consisted of a black Plexiglas maze with three arms of equal length (120° angles between arms). During the task, each rat was placed in the center of the maze and allowed to explore freely for 8 min. An alternation was defined as three consecutive entries into different arms in overlapping triplet sequences (e.g. entering arms A, B, and C in any order without repetition). The alternation percentage was calculated as follows:

Alternation (%) = (​$\frac{\mathrm{Number of alternations}}{\mathrm{Total arm entries}-2}$) × 100

Arms were disinfected with 10% ethanol between examinations to minimize olfactory cues impact.

### Novel object recognition (NOR) task

This task assesses NOR memory by examining the disparities in the exploration durations of familiar and unfamiliar objects. After an initial 5-min adaptation, each rat underwent two 5-min object exploration trials, with a 4-hr inter-trial interval. The objects were unfamiliar to the animals. During the initial (familiarization) trial, the rats explored two identical red plastic cubic boxes measuring 6x6x6 cm each. In the subsequent testing session, one object was substituted with a new blue-colored metal cylinder, 6 cm in diameter and 6 cm in altitude. Object placements were counterbalanced across all experimental groups to mitigate potential biases arising from rodents' preferences for specific objects or positions. Object exploration including behaviors such as chewing, sniffing, licking, or approaching within ≤1 cm of the object, was observed. The discrimination ratio was calculated using the formula: 

(t [novel]/t [total for familiar and novel]) * 100.

### Passive avoidance test

This task estimates rodents' memory ability and conditioned learning. The rodents underwent testing within a shuttle box containing two dark and illuminated chambers connected by a door and a grid floor. Rats were allowed to get used to the apparatus for five minutes on the 18th and 19th days post-surgery. The rodents performed an acquisition trial within the shuttle box on the twentieth day. The door was raised, the light was activated, and the first latency, representing the duration of entering the dim room, was determined after five minutes. The door descended as the rodent accessed the dim compartment, and the animal was subjected to a mild foot electrical shock of 1 mA for 1 second. The following day, the test was replicated. Each rat was put in the illuminated room, and the durations taken to move from the dimly lit area were measured as the step-through latencies.

### Assessment of oxidative stress, neuroinflammation, and apoptosis in the hippocampus

Three weeks post-amyloid β microinjection, hippocampal tissues (n = 6 per group) were extracted from deeply anesthetized rodents. Tissues underwent washing with ice-cold PBS. Then, 5% homogenates were produced in an icy-cold Tris-HCl buffer solution (150 mM) supplemented with a mixture of protease inhibitors from SigmaAldrich, USA (Cat # P8340). Following centrifugation at 5000 rpm at 4°C, the supernatant solution was isolated for subsequent tests.

The thiobarbituric acid method was employed to quantify the tissue concentration of malondialdehyde (MDA) as a marker of lipid peroxidation and oxidative stress (Baluchnejadmojarad et al. 2017). The outcome is presented in nmol/mg of protein, with tetraethoxypropane serving as the standard reference substance.

The evaluation of superoxide dismutase (SOD) activity as an antioxidant enzyme involved incubating supernatant solutions with xanthine oxidase and xanthine for 40 min before adding nitroblue tetrazolium. The production of blue formazan was observed at a wavelength of 550 nm.

Nitrite levels as an indicator of nitric oxide metabolism were assessed using the Griess method (Wopara et al. 2021). In this experimental protocol, the supernatants were exposed to a Griess reagent comprising sulfanilamide and naphthyl ethylenediamine (both from Sigma-Aldrich, USA) in an acidic medium.

The hippocampal level of TNFα as a pro-inflammatory cytokine was determined using an ELISA kit from Sigma-Aldrich, USA (Cat # RAB0480) and in accordance with the ELISA sandwich method. The absorbing capacity of microwells was quantified at 450 nm using a single-channel microplate reader, and the results are expressed as concentrations (w/v) or fold changes relative to the control experimental group.

The enzymatic activity of caspase-3 as a measure of apoptosis was assessed following established protocols (Baluchnejadmojarad et al. 2019). The assay kit (Cat # ab39401) relied on the hydrolysis of p-nitroaniline (pNA) substrate by caspase-3. Tissue samples were incubated with an enzyme measurement buffer containing sucrose, 3-[(3-Cholamidopropyl)dimethylammonio]-1-propanesulfonate hydrate (CHAPS), 4-(2-Hydroxyethyl)-1-piperazine ethanesulfonic acid (HEPES), dithiothreitol, Ethylenediaminetetraacetic acid (EDTA), and caspase-3 apopain substrate, which is a specific chromogenic pNA substrate. The release of fluorescent pNA was measured with a microplate reader (BioTek, USA) at an absorbance wavelength of 405 nm. The results are presented as the mean optical density of the experimental groups.

### Measurement of AChE activity in the hippocampus

The measurement of hippocampal AChE activity as a marker of cholinergic function was conducted using a modified Ellman method (Isomae et al. 2003) and by its specific kit from SigmaAldrich, USA (Cat # CS0003). Briefly, AChE activity was detected by evaluating the generation of a yellow product resulting from the reaction between thiocholine, generated through the enzymatic breakdown of acetylthiocholine, and Ellman’s reagent at a wavelength of 412 nm, following the protocol provided by Abcam, USA.

### Measurement of hippocampal levels of synaptophysin and Microtubule-associated protein 2

The levels of synaptophysin as a synaptic factor (MyBioSource, USA; Cat # MBS2086975) and Microtubule-associated protein 2 (MAP2) as a neuronal integrity and dendritic marker (MyBioSource, USA; Cat # MBS704489) were quantified using the sandwich ELISA technique in the tissue homogenates.

### Histological evaluation of the hippocampus

Transcardial perfusion was conducted using 50 ml of heparinized N/S 0.9%, followed by 50–75 ml of a fixative buffer comprising 4% paraformaldehyde in 0.1 M PBS. The rat brains (n = 5 per group) were extracted and subjected to post-fixation for a duration of one week. The left hippocampal blocks were meticulously processed and encased in paraffin wax. The hippocampal blocks were precisely sectioned into coronal sections (7 μm) and subjected to H&E staining. The ratio of dead/total pyramidal neurons in percentage was determined in the CA1 area of the hippocampus. It was quantified in a minimum of 4 sections ranging from −3.6 to −4.3 millimeters from the bregma, within a 0.1 mm^2^ region, utilizing the image acquisition and analysis system provided by Bel Engineering Co., Italy. Cells exhibiting well-defined cytoplasmic boundaries and a prominent nucleolus were considered in the counting process. Each section underwent the counting process three times, conducted in a blinded manner. 

### Statistical analysis

The values are expressed as the mean ± SEM. Data normality was validated by the Kolmogorov-Smirnov test, followed by an ANOVA test to identify differences between experimental groups. If significant differences were observed, pairwise comparisons were performed using the Tukey post-hoc test. The significance levels were established at p<0.05. Statistical analyses were conducted using GraphPad Prism, version 8.4.3.

## Results

### Behavioral findings

**Figure 2:**
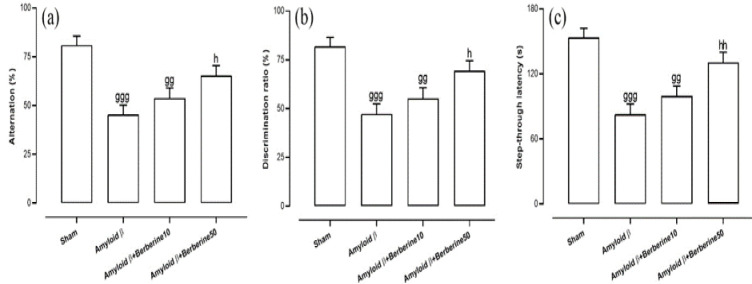
*Behavioral performance in the Y-maze test (a), NOR task (b), and passive avoidance test (c). Panel (a) shows the alternation percentage in the Y-maze test, with higher percentages indicating better cognitive performance. Panel (b) presents the discrimination ratios in the NOR task, assessing memory and recognition ability. Panel (c) shows the step-through latencies in the passive avoidance test, with longer latencies reflecting improved memory retention.* *Significant differences: gg (p* *<0.01), and* *ggg (p* *<0.* *001) vs. sham; h (p* *<0.05), and* *hh (p* *<0.01) vs. amyloid β group.*

Figure 2a illustrates the Y-maze task outcomes, assessing short-term working spatial recognition recall. Our analysis showed significant differences among the tested groups (F(3,28)=9.58, p<0.001). Analysis of results by Tukey post-test showed that the alternation percentage in the amyloid β group was significantly lower relative to the sham group (p<0.001). In addition, alternation was significantly lower in amyloid β + berberine 10 group versus the sham group (p<0.01) and such significant reduction was not observed in amyloid β + berberine 50 group when compared to the sham group (p>0.05). In addition, alternation score in the amyloid β + berberine 10 group was not significantly greater as compared to the amyloid β group (p>0.05). In contrast, alternation score in the amyloid β + berberine 50 group was significantly higher when compared to the amyloid β group (p<0.05). Furthermore, the locomotor activity of the animals was measured by counting the total number of times they entered the maze. The results showed that this parameter does not differ significantly among the groups (p>0.05) (data not presented).

The NOR task was utilized to assess the animals' cognitive abilities in recognition memory. Our analysis revealed significant differences among the groups (F(3,28)=8.95, p<0.001). The Tukey post-hoc test showed that the discrimination ratio was significantly lower in the amyloid β group compared to the sham group (p < 0.001). Additionally, a significant reduction was observed in the amyloid β + berberine 10 group versus the sham group (p<0.01), whereas such a reduction was not observed in the amyloid β + berberine 50 group when compared to the sham group (p>0.05). Furthermore, the discrimination ratio in the amyloid β + berberine 50 group was significantly higher than that in the amyloid β group (p<0.05) (Figure 2b).

The passive avoidance test was utilized to evaluate the conditional learning and memory abilities (Figure 2c). Data analysis showed significant differences among the tested groups (F(3,28)=11.90, p<0.001). The Tukey post-hoc test revealed that step-through latency was significantly lower in the amyloid β group compared to the sham group (p < 0.001). A significant reduction was also observed in the amyloid β + berberine 10 group versus the sham group (p < 0.01), whereas such a reduction was not observed in the amyloid β + berberine 50 group when compared to the sham group (p>0.05). Furthermore, the step-through latency in the amyloid β + berberine 50 group was significantly higher than that in the amyloid β group (p<0.01).

### The levels and/or activity of biomarkers associated with oxidative stress, neuroinflammation, and apoptosis in the hippocampus

**Figure 3:**
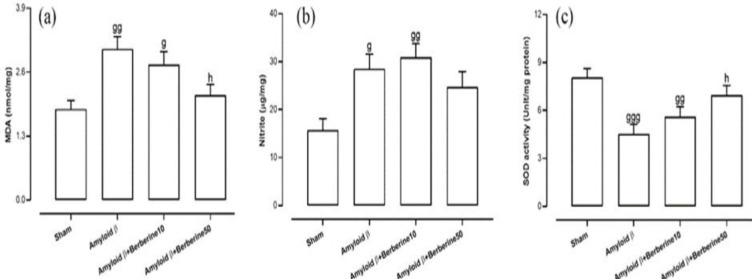
Oxidative and nitrosative stress markers in the hippocampus. Panels display MDA level (a), nitrite level (b), and SOD activity (c), indicating lipid peroxidation, nitrosative stress, and antioxidant capacity, respectively. Significant differences: g (p<0.05), gg (p<0.01), and ggg (p<0.001) vs. sham; h (p<0.05), and hh (p<0.01) vs. amyloid β group.

Regarding oxidative stress-related biomarkers, one-way ANOVA analyses showed significant intergroup differences in hippocampal levels of MDA (F(3,24) = 5.87, p<0.01), nitrite (F(3,24) = 5.23, p<0.01), and SOD activity (F(3,24) = 6.31, p<0.01). Post-hoc comparisons revealed that the amyloid β group exhibited a significant increase in MDA (p<0.01) (Figure 3a) and nitrite levels (p<0.05) (Figure 3b), along with a significant decrease in SOD activity (p<0.001) (Figure 3c), compared to the sham group. Similarly, the amyloid β + berberine 10 group showed significantly elevated levels of MDA (p<0.05) and nitrite (p<0.01), as well as a reduced SOD activity (p<0.01), relative to the sham group. Treatment with berberine at a dose of 50 mg/kg in amyloid β-injected rats significantly attenuated MDA levels (p<0.05) and improved SOD activity (p<0.05) when compared to the amyloid β group. Although a reduction in nitrite levels was also observed in the amyloid β + berberine 50 group, the difference did not reach statistical significance (p>0.05).

**Figure 4:**
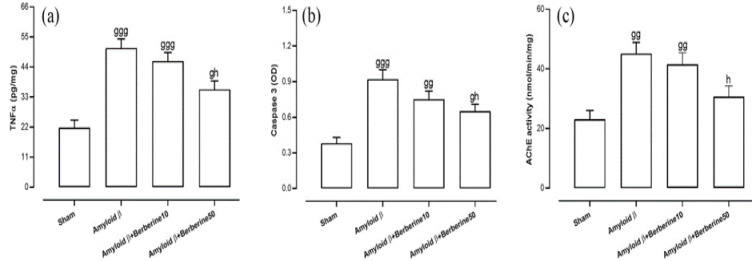
*Levels of TNFα (a), caspase-3 activity (b), and AChE activity (c) in the hippocampus, reflecting inflammation, apoptosis, and cholinergic dysfunction, respectively* *. Significant differences: g (p* *<* *0.05), gg (p* *<0.01), and* *ggg (p* *<* *0.001) vs. sham; h (p* *<0.05) vs. amyloid β group.*

The concentration of TNFα in the hippocampus was quantified as a marker of neuroinflammation. One-way ANOVA revealed significant differences among the experimental groups (F(3,24) = 17.14, p<0.001). Post-hoc analysis indicated that the amyloid β group exhibited a markedly elevated TNFα concentration compared to the sham group (p<0.001) (Figure 4a). Similarly, both the amyloid β + berberine 10 (p<0.001) and amyloid β + berberine 50 (p<0.05) groups showed significantly increased TNFα levels relative to the sham group. However, TNFα concentration in the amyloid β + berberine 50 group was significantly lower than that observed in the amyloid β group (p<0.05).

Regarding the apoptotic biomarker, one-way ANOVA revealed significant intergroup differences in hippocampal caspase-3 levels (F(3,24) = 11.75, p<0.001). Post-hoc analysis showed that the amyloid β group exhibited a marked increase in caspase-3 levels compared to the sham group (p < 0.001) (Figure 4b). The amyloid β + berberine 10 group also demonstrated a significant elevation in caspase-3 levels relative to the sham group (p<0.01), and a similar but less pronounced increase was observed in the amyloid β + berberine 50 group (p<0.05). However, treatment with berberine at a dose of 50 mg/kg significantly reduced caspase-3 levels in comparison to the amyloid β group (p<0.05).

### Hippocampal levels of AChE activity

 Statistical analysis using one-way ANOVA demonstrated a significant variation in hippocampal AChE levels across the experimental groups (F(3,24) = 7.94, p<0.001). Post-hoc analysis indicated that the amyloid β group exhibited a significant increase in AChE levels compared to the sham group (p<0.01) (Figure 4c). Similarly, the amyloid β + berberine 10 group showed a significant elevation in AChE levels relative to the sham group (p<0.01). In contrast, the amyloid β + berberine 50 group demonstrated a significant reduction in AChE levels compared to the amyloid β group (p<0.05).

### Hippocampal levels of synaptic plasticity-related biomarkers

**Figure 5:**
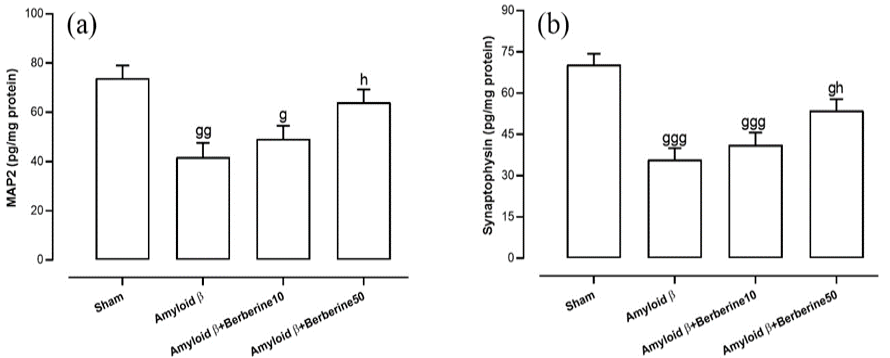
*Synaptic markers in the hippocampus. Panels show MAP2 (a) and synaptophysin (b) levels, indicating dendritic stability and synaptic function* *. Significant differences: g (p* *<* *0.05), gg (p* *<0.01), and* *ggg (p* *<* *0.001) vs. sham; h (p* *<0.05) vs. amyloid β group.*

Levels of MAP2 and synaptophysin were evaluated as biomarkers associated with synaptic plasticity. One-way ANOVA revealed significant differences in hippocampal MAP2 levels among the experimental groups (F(3,24) = 7.36, p<0.01) (Figure 5a). Post-hoc analysis showed that MAP2 levels were significantly reduced in the amyloid β group compared to the sham group (p<0.01). A similar, though less pronounced, reduction was observed in the amyloid β + berberine 10 group (p<0.05). In contrast, MAP2 levels in the amyloid β + berberine 50 group did not differ significantly from those in the sham group (p>0.05). Moreover, treatment with berberine at a dose of 50 mg/kg in amyloid β-injected rats significantly increased MAP2 levels relative to the amyloid β group (p<0.05).

Additionally, one-way ANOVA revealed significant differences in hippocampal synaptophysin levels among the experimental groups (F(3,24) = 13.52, p<0.001) (Figure 5b). Post-hoc analysis showed that synaptophysin levels were significantly reduced in the amyloid β group compared to the sham group (p<0.001). A similar reduction was observed in the amyloid β + berberine 10 group, which also showed significantly lower synaptophysin levels relative to the sham group (p<0.001). In contrast, synaptophysin levels in the amyloid β + berberine 50 group were not significantly different from those in the sham group (p>0.05). Furthermore, administration of berberine at a dose of 50 mg/kg significantly elevated synaptophysin levels compared to the amyloid β group (p<0.05).

### Histological findings

**Figure 6:**
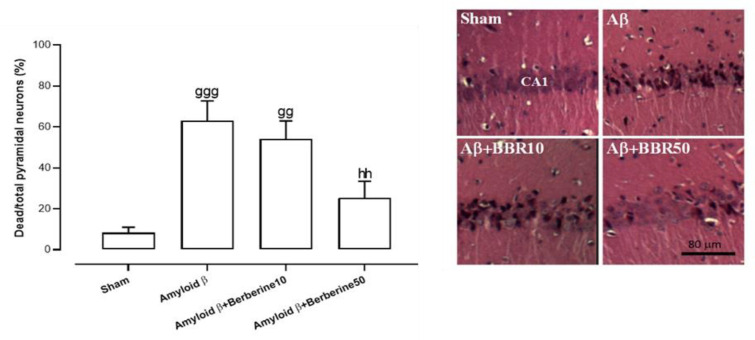
*Ratio of dead/total pyramidal neurons in the CA1 hippocampal region. Panel (a) quantifies this ratio for H&E-stained sections; panel (b) displays photomicrographs showing such changes. BBR refers to berberine, and Aβ refers to amyloid β.* *Significant differences: gg (p* *<0.01), and* *ggg (p* *<* *0.001) vs. sham; hh (p* *<0.01) vs. amyloid β group*

The ratio of dead/total pyramidal neurons in the CA1 field of the hippocampus in the amyloid β group was significantly higher than in the sham group at p<0.001 (ANOVA Table: F(3,16)=10.72, p<0.001). A similar significant increase was also obtained to a lesser extent in the amyloid β + berberine 10 group (p<0.01). In addition, the berberine intervention at a dose of 50 mg/kg significantly ameliorated this increase in the CA1 field of the hippocampus in the amyloid β-injected rats (p<0.01) and amyloid β + berberine 10 group did not show significant change regarding this factor relative to the amyloid β group (p>0.05) (Figure 6).

## Discussion

Despite significant recent advancements in AD research, only symptomatic treatments are currently available for this condition (Passeri et al. 2022). Several natural drugs have demonstrated important neuroprotective, antioxidant, and anti-inflammatory properties and are effective at treating multiple neurodegenerative diseases (Sarkar et al. 2024). However, this study highlights the unique effects of berberine in mitigating amyloid β-induced damage in the hippocampus, showcasing its potential mechanisms of action which have not been fully explored previously. *B. vulgaris* has been utilized in traditional medicinal practices, with its noteworthy neuroprotective properties. The plant contains a diverse array of isoquinoline alkaloids, predominantly concentrated in the root structures. Berberine, as the most extensively investigated and contested constituent of *B. vulgaris*, has exhibited neuroprotective effects in various neurodegenerative models of rodents, especially at a dose of 50 mg/kg/d (Gasmi et al. 2024).

The extracellular accumulation of amyloid plaques represents a prevalent histopathological observation in AD. Previous studies have linked intrahippocampal amyloid β microinjection to impairments in learning and memory functions (Carles et al. 2024). In this study, amyloid β-induced memory and learning disruptions were evident in behavioral tests, including the Y-maze, NOR, and passive avoidance tasks. Our findings confirmed that berberine, particularly at 50 mg/kg/day, significantly improved these cognitive impairments, emphasizing its potential for therapeutic use. The results were consistent with the study by de Oliveira et al. (de Oliveira et al. 2016), which indicated that berberine improves rats' cognitive memory performance in behavioral tests.

There is research evidence demonstrating the incremental and negative effects of oxidative stress throughout the development of AD (Bisht et al. 2018). In this investigation, the levels/activity of oxidative stress biomarkers in the hippocampus exhibited a decrease by the end of the third week after intrahippocampal microinjection of amyloid β_1-42_, aligning with prior research results (Baluchnejadmojarad et al. 2019). Conversely, consistent with previous studies (Sadraie et al. 2019), berberine at a dosage of 50 mg/kg/day, but not at 10 mg/kg/day, reduced the intensity of hippocampus oxidative-nitrosative stress. The potential neuroprotective mechanism of berberine may involve the stimulation of the Nrf2 signaling pathway, which is recognized for its role in controlling the expression of antioxidant enzymes such as SOD. The increase in SOD activity noted in this investigation may directly result from berberine capacity to stimulate this pathway, thus alleviating oxidative stress and averting neuronal damage. Moreover, the involvement of berberine in modulating mitochondrial function may also contribute to its antioxidant effects by enhancing mitochondrial biogenesis and reducing mitochondrial dysfunction commonly seen in AD (Tian et al. 2023).

Chronic inflammation in AD is histopathologically characterized by activated microglia, reactive astrocytes, and elevated inflammatory cytokine release. Microglia secrete TNFα in response to stimulation with amyloid β, which in turn, both reduces microglial phagocytosis of amyloid β and upregulates the production of secretases, further driving amyloidogenesis (Wang et al. 2015). Our findings demonstrated that TNFα levels increased following amyloid β injection, consistent with earlier results (Gao et al. 2022), while berberine effectively reduced this inflammatory response at 50 mg/kg/day dose, consistent with previous studies (Hussien et al. 2018). The anti-inflammatory effect of berberine in this study can be attributed to its ability to inhibit the NF-κB pathway, a critical regulator of pro-inflammatory cytokine production. By suppressing TNFα and other cytokines, berberine may not only reduce neuroinflammation but also protect against neurodegeneration by modulating microglial activation and improving amyloid β clearance (Tian et al. 2023).

Elevated levels of hippocampal caspase-3 are identified as a potential apoptotic biomarker associated with an unfavorable prognosis in neurodegenerative conditions (Pérez et al. 2024). Nevertheless, divergent findings exist regarding the changes in apoptosis following hippocampal administration of amyloid β, with some suggesting that amyloid β did not markedly elevate apoptotic markers (Chen and Dong 2009), while others indicated its up-regulative effects (Boland and Campbell 2004), consistent with our study. In this study, berberine significantly attenuated caspase-3 activity in amyloid β-injected rats, aligning with its known anti-apoptotic properties. These results are supported by previous studies on the anti-apoptotic properties of this isoquinoline compound (Alonso Bellido et al. 2023). The anti-apoptotic effect of berberine might be linked to its modulation of the Bcl-2 family proteins and the inhibition of the caspase cascade. Berberine interaction with these pathways could help to prevent mitochondrial dysfunction and cell death by restoring mitochondrial integrity, a critical factor in the pathogenesis of AD (Tian et al. 2023).

Dysfunction in the CNS cholinergic system is linked to disruptions in learning and memory functions (Ferreira-Vieira et al. 2016). In this study, there was increased activity of AChE in the brain of rats challenged with amyloid β, and berberine mitigated this alteration. In line with our findings, prior research has also demonstrated that berberine can exert its neuroprotective properties through AChE inhibition (Hussien et al. 2018).

Synaptophysin, as a synaptic plasticity and transmission marker, has been shown to be involved in the AD rat models and is associated with cognitive function in AD (Sze et al. 1997). Our findings demonstrated a significant reduction in synaptophysin levels in amyloid β-injected rats, which was reversed by berberine treatment, suggesting its protective effect on synaptic integrity. Supporting the current findings, previous studies have shown that berberine can boost synaptophysin expression and reduce amyloid β pathogenesis (Cai et al. 2019). Berberine-induced restoration of synaptophysin expression suggests that it may promote synaptic plasticity by stimulating pathways involved in synaptic vesicle recycling and neurotrophin signaling. The compound may also reduce amyloid β-induced synaptic dysfunction through the activation of brain-derived neurotrophic factor (BDNF) or the inhibition of tau phosphorylation, both of which play crucial roles in synaptic integrity (Mohseni et al. 2023; Tian et al. 2023).

MAP2 is a protein highly concentrated in dendrites and it functions as an indicator of synaptic plasticity. Amyloid β accumulation in neurons is spatially related to MAP2 dysfunction, suggesting a relationship between amyloid β_1-42_ accumulation and MAP2 reduction (Zhao et al. 2019). Berberine intervention in this study successfully prevented MAP2 reduction, further supporting its role in preserving dendritic integrity in amyloid β-induced neurodegeneration. Naveen et al. demonstrated similar outcomes, suggesting that berberine enhances neuronal differentiation markers and effectively inhibits the decline in MAP2 (Naveen et al. 2016). 

The present study demonstrated a notable neuronal loss in the CA1 region of the hippocampus within the amyloid β group relative to the sham-operated group. Corbett et al. indicated a 40% loss of pyramidal cells in the dorsal CA1 after amyloid β intracerebroventricular injection (Corbett et al. 2013). In the present investigation, the administration of berberine at a dosage of 50 mg/kg resulted in a substantial increase in both the quantity and density of pyramidal neurons in comparison to the amyloid β group. Consistent with these findings, Aski et al. demonstrated that berberine chloride (50 mg/kg) ameliorates hippocampal neuronal depletion in a rat model of vascular dementia (Aski et al. 2018).

The main constraints of this study include the lack of evaluation of the long-term effects and the persistence of berberine neuroprotective benefits beyond the experimental period, which limits our understanding of its prolonged therapeutic potential. Additionally, the study did not perform a comprehensive correlation analysis between the various biochemical markers and behavioral outcomes, which could have provided deeper insights into the interrelationships between these factors. Furthermore, the study did not explore the effects of alternative administration methods of berberine, which could offer further clarity on its optimal therapeutic regimen. These aspects could be addressed in future research.

 The results of this investigation demonstrated that berberine exhibits a dose-dependent and partial protective effect against amyloid β-induced cognitive impairments, as evidenced by the enhanced performance of rats in Y-maze, NOR, and passive avoidance tasks. The neuroprotective and therapeutic effects of berberine potentially contribute to its impact on hippocampal inflammation, oxidative and nitrosative stress, apoptosis, synaptic protection, and inhibition of AChE activity. These findings highlight berberine as a promising candidate for neuroprotection in AD. Future research should focus on further elucidating its mechanisms of action and evaluating its therapeutic potential in clinical settings.
